# C-reactive protein-albumin-lymphocyte (CALLY) index predicts overall survival in elderly Japanese patients with dysphagia: a retrospective cohort study

**DOI:** 10.3389/fnut.2025.1681956

**Published:** 2025-10-13

**Authors:** Wenxiu Pan, Yawen Yang, Xiaohui Song, Jixian Wang, Qing Xie

**Affiliations:** ^1^Department of Rehabilitation Medicine, Ruijin Hospital, School of Medicine, Shanghai Jiao Tong University, Shanghai, China; ^2^Department of Rehabilitation Medicine, Shanghai Ruijin Rehabilitation Hospital, Shanghai, China

**Keywords:** CALLY, C-reactive protein-albumin-lymphocyte index, dysphagia, survival, prognosis

## Abstract

**Background:**

The C-reactive protein-albumin-lymphocyte (CALLY) index serves as an established prognostic biomarker across multiple severe disease cohorts. Nevertheless, limited research has examined its relationship with overall survival among elderly Japanese individuals experiencing dysphagia.

**Objective:**

To evaluate the prognostic utility of the CALLY index for overall survival in a cohort of elderly Japanese patients with dysphagia.

**Methods:**

We conducted a retrospective single-center cohort study of 248 patients diagnosed with dysphagia between January 2014 and January 2017. The primary outcome was overall survival. The CALLY index was analyzed both continuously (natural log transformation) and by quartiles. Multivariable Cox proportional hazards models adjusted for relevant demographic, clinical, and nutritional covariates (including age, sex, comorbidities, hemoglobin, feeding modality, and daily caloric intake) were used to estimate hazard ratios (HR) and 95% confidence intervals (CI). Restricted cubic spline models assessed dose–response relationships. Kaplan–Meier analysis estimated median survival by CALLY quartile; subgroup analyses examined effect modification.

**Results:**

After natural log transformation, the CALLY index was independently associated with improved survival (HR = 0.85, 95%CI: 0.76–0.95, *p* = 0.003). Using the lowest quartile (Q1) as reference, adjusted HR were 0.73 (95%CI: 0.46–1.16, *p* = 0.179) for Q2, 0.56 (95%CI: 0.34–0.90, *p* = 0.018) for Q3, and 0.44 (95%CI: 0.25–0.78, *p* = 0.005) for Q4. Restricted cubic spline analysis indicated a positive, approximately linear relationship between the CALLY index and overall survival. Median survival times were 887, 785, 362, and 153 days for Q4, Q3, Q2, and Q1, respectively. Subgroup analyses showed no significant interactions across prespecified strata.

**Conclusion:**

In this cohort of elderly Japanese patients with dysphagia, a higher CALLY index was associated with longer overall survival. These findings support the potential prognostic utility of the CALLY index in this population; prospective and multicentre validation is warranted.

## 1 Introduction

Dysphagia is common among older adults. Advancing age represents a well-established risk factor, with octogenarians exhibiting particularly strong associations between dysphagia and multisystem morbidity (1). Affected individuals commonly develop secondary complications including malnutrition, pneumonia, and dehydration. These sequelae contribute to prolonged hospitalization, elevated healthcare expenditures, and diminished quality of life (2–4). Consequently, prioritizing dysphagia assessment and management is of paramount clinical importance. Evidence indicates that both solitary nutritional indicator and composite inflammation–nutrition indices predict the incidence of mortality in older adults with dysphagia (5, 6). The CALLY index integrates systemic inflammation (C-reactive protein), nutritional reserve (serum albumin), and immune competence (lymphocyte count), thereby capturing multiple pathophysiological domains that are highly relevant to outcomes in dysphagia (7). Originally devised as a nutrition-immunity composite score for hepatocellular carcinoma (7, 8), the CALLY index has since become a robust prognostic marker in oncology (9–15). More recently, it has also been shown to possess prognostic value in non-tumor conditions (16–22).

However, its prognostic performance in geriatric populations with dysphagia has not been established. We therefore evaluated whether the CALLY index independently predicts overall survival in an elderly Japanese cohort with dysphagia and whether it offers potential advantages over conventional single biomarkers.

## 2 Materials and methods

### 2.1 Study design

This Japanese single-center retrospective cohort study performed secondary analysis of anonymised Dryad datasets. Elderly participants consecutively enrolled from January 2014 to January 2017 were diagnosed with clinically verified dysphagia. Diagnostic confirmation involved multidisciplinary assessment by physicians, nurses, and speech-language pathologists, with universal videofluoroscopy demonstrating significant swallowing dysfunction. Nutritional interventions comprised: (a) Percutaneous endoscopic gastrostomy (PEG) using modified introducer technique, and (b) Total parenteral nutrition (TPN) delivered through implantable ports (PORT), non-tunneled central venous catheters (NT-CVC), or peripherally inserted central catheters (PICC). The dataset incorporated age, sex, cerebrovascular disease, severe dementia, aspiration pneumonia, ischaemic heart disease, nutritional support approach (PEG/TPN), oral feeding resumption status, laboratory biomarkers, and daily caloric consumption (kcal/day). Venous blood sampling was conducted within 7 days preceding nutritional intervention commencement. Exclusion criteria covered advanced malignancies, PEG for gastric decompression, and pre-2014 PEG placements among TPN recipients. Patients receiving combination therapy were assigned to the PEG cohort.

### 2.2 Data sources

Study data were sourced from Dryad Digital Repository (23), an open-access platform for unrestricted retrieval of primary datasets. The initial data acquisition was conducted ethically, and the subsequent reuse of these data adheres to open-data guidelines.

### 2.3 Variables

Interdisciplinary teams collaboratively determined the enteral versus parenteral nutrition route with patients or their legally authorized representatives. Standardized clinical assessment protocols guided nutritional intervention implementation by healthcare providers. Patient-level clinical metrics were retrospectively extracted from medical records, encompassing age, sex, comorbidities, preprocedural laboratory parameters, and nutritional intake metrics. Baseline Clinical Frailty Scale (CFS) were obtained at the time of PEG tube or TPN catheter insertion. Hematological assessments conducted within 7 days preceding nutritional support initiation were analyzed, including serum albumin, hemoglobin, C-reactive protein, and lymphocyte count. Caloric intake was quantified on post-procedure day 7 across both nutritional cohorts. Clinical diagnoses of severe pneumonia and sepsis were established by trained physicians. Functional oral intake recovery required more than 30-day cessation of artificial feeding. Survival status and follow-up duration were prospectively recorded for each dysphagia case. Overall survival duration was defined as the period from hospital admission to mortality or last documented follow-up.

The CALLY index is computed using:

$\left[\mathrm{Alb}\;(g/\mathrm{dL})\times \text{Lymphocyte count}\;({\mathrm{mm}}^{3})]/[\mathrm{CRP}\;(\mathrm{mg}/\mathrm{dL})\times 10\right]$

### 2.4 Statistical analysis

Secondary analyses employed publicly available datasets. Given that missing values accounted for <5% of observations, we considered complete case analysis appropriate and therefore excluded records with missing data from the primary analyses. Categorical variables are presented as percentages (%), continuous variables as mean ± standard deviation or median [interquartile range]. Given the left-skewed distribution of CALLY values, natural logarithmic transformation preceded analytical procedures (9). Baseline characteristics were compared across CALLY quartiles using one-way ANOVA for continuous measures and chi-square tests for categorical variables. Cox proportional hazards regression evaluated the association between CALLY index and overall survival in dysphagia patients. To assess the Cox proportional hazards assumption, we used graphical methods (log–log) survival plots and plots of scaled Schoenfeld residuals. If the assumption was rejected, we considered a stratified Cox model for the relevant variable(s) or inclusion of time-dependent interaction terms in the model (24). We constructed a total of four models to evaluate the relationship between CALLY index and mortality. Model 1 was adjusted for age and sex. Model 2 included additional adjustments for cardiovascular disease (CVD), dementia, ischemic heart disease (IHD), hemoglobin, and aspiration pneumonia (Asp). Model 3 further adjusted for the type of nutrition (PEG, TPN, oral intake recovery). Model 4 included additional adjustments for daily caloric intake, and served as the primary model which included all previously mentioned adjustments. Kaplan–Meier curves with log-rank tests depicted survival probability. Likelihood ratio tests examined subgroup interactions. In addition, restricted cubic spline (RCS) regression was performed with 4 knots at the 5th, 35th, 65th, and 95th percentiles (Harrell’s recommendation) of CALLY index to assess non-linearity and examine the dose–response curve between CALLY index and overall survival after adjusting variables in Model 4. All analyses were conducted using R 4.2.2 (The R Foundation)^1^ and Free Statistics software version 2.2 (25). Statistical significance was defined as two-tailed *p* < 0.05.

## 3 Results

### 3.1 Baseline characteristics of the participants

**Figure 1:**
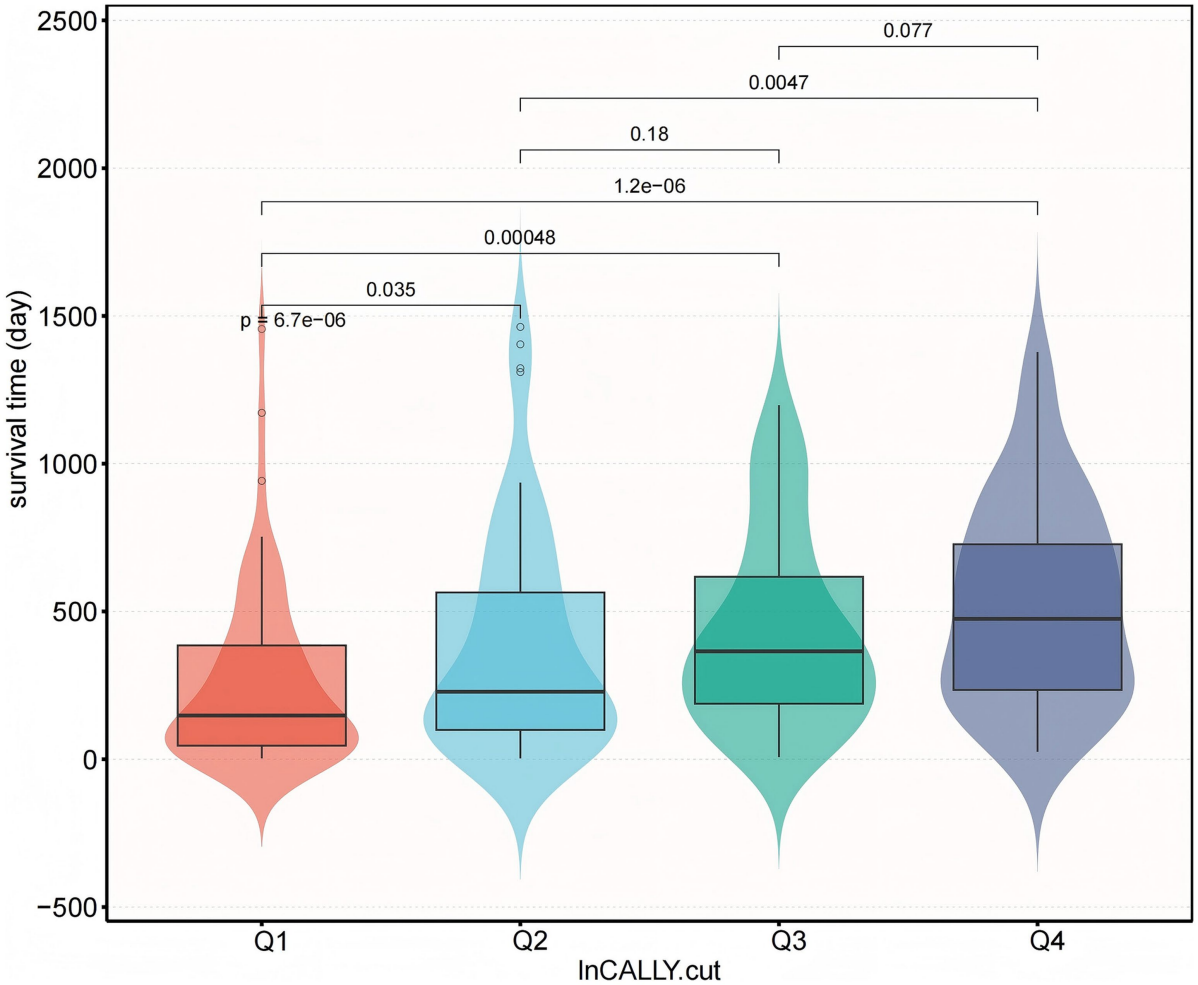
Survival time distribution by ln-transformed CALLY index quartiles. Combined boxplot (median, interquartile range, whiskers: min-max excluding outliers) and violin plot (kernel density estimation) demonstrating significant differences in survival time (days) across quartiles of natural log-transformed CALLY index. Q1 represents the lowest ln-CALLY quartile (red), Q2 (light blue), Q3 (green), and Q4 the highest quartile (dark blue). Survival time progressively increased with ascending CALLY quartiles (*p* < 0.001 for trend), with pairwise comparisons showing significantly shorter survival in Q1 versus Q2 (*p* = 6.7 × 10^−6^), Q3 (*p* = 0.0047), and Q4 (*p* = 0.077, non-significant). All statistical comparisons performed using Kruskal–Wallis with Dunn’s post-hoc.

Following exclusion of individuals with five incomplete lymphocyte data, the final analytical cohort included 248 patients with dysphagia (mean age 83 years, standard deviation 9.3; 39.1% male). The baseline characteristics of excluded versus included patients were shown in Supplementary Table S1. The overall survival rate reached 46%. Table 1 displays baseline patient characteristics categorized according to ln-CALLY index quartiles. Significant variation in survival duration was evident among these quartiles (*p* < 0.05) in Figure 1. Lower CALLY index values demonstrated a statistical significant link to older age, decreased hemoglobin concentrations, reduced daily caloric intake and decreased PEG utilization (*p* < 0.05). History of severe dementia, Asp., PEG use, and daily caloric intake exhibited statistically significant differences between groups (*p* < 0.05). In contrast, no significant intergroup variations (*p* > 0.05) were found regarding sex distribution, CVD history, TPN administration, or oral intake resumption.

**Table 1:** Baseline characteristics of patients with dysphagia.

Variables	Total	Q1 (≤ − 0.168)	Q2 (−0.168 to 1.38)	Q3 (1.38–2.84)	Q4 (>2.84)	*p*-value
	(*n* = 248)	(*n* = 62)	(*n* = 62)	(*n* = 62)	(*n* = 62)	
Age (years), Mean ± SD	83.0 ± 9.3	85.1 ± 6.8	84.1 ± 8.1	82.2 ± 10.6	80.8 ± 10.7	0.049
Sex, *n* (%)						0.223
Male	97 (39.1)	29 (46.8)	26 (41.9)	24 (38.7)	18 (29)	
Female	151 (60.9)	33 (53.2)	36 (58.1)	38 (61.3)	44 (71)	
CVD, *n* (%)	132 (53.2)	25 (40.3)	33 (53.2)	34 (54.8)	40 (64.5)	0.061
Dementia, *n* (%)	100 (40.3)	32 (51.6)	29 (46.8)	23 (37.1)	16 (25.8)	0.018
Asp., *n* (%)	93 (37.5)	30 (48.4)	30 (48.4)	20 (32.3)	13 (21)	0.003
IHD, *n* (%)	44 (17.7)	17 (27.4)	11 (17.7)	8 (12.9)	8 (12.9)	0.113
Hemoglobin (g/dl), Mean ± SD	11.0 ± 2.0	9.9 ± 1.9	10.9 ± 2.0	11.0 ± 2.0	12.2 ± 1.6	<0.001
Daily calorie intake (kcal), Mean ± SD	917.6 ± 187.1	870.8 ± 237.3	895.8 ± 168.2	940.8 ± 166.1	962.9 ± 155.6	0.024
Oral, *n* (%)	14 (5.6)	2 (3.2)	2 (3.2)	3 (4.8)	7 (11.3)	0.202
PEG, *n* (%)	180 (72.6)	36 (58.1)	41 (66.1)	47 (75.8)	56 (90.3)	<0.001
TPN, *n* (%)	73 (29.4)	16 (25.8)	19 (30.6)	16 (25.8)	22 (35.5)	0.589
Status, *n* (%)						<0.001
Alive	114 (46.0)	14 (22.6)	24 (38.7)	33 (53.2)	43 (69.4)	
Dead	134 (54.0)	48 (77.4)	38 (61.3)	29 (46.8)	19 (30.6)	
CALLY, Median (IQR)	4.0 (0.8, 17.2)	0.4 (0.2, 0.6)	2.0 (1.4, 2.8)	8.0 (5.6, 12.3)	42.3 (25.5, 82.5)	<0.001

PEG, percutaneous endoscopic gastrostomy; TPN, total parenteral nutrition; CVD, cerebrovascular diseases; Dementia, severe dementia; Asp, aspiration pneumonia; IHD, ischemic heart diseases; Oral, oral intake recovery; CALLY, CRP-albumin-lymphocyte index.

### 3.2 Prognostic significance of the CALLY index in elderly dysphagia patients

**Figure 2:**
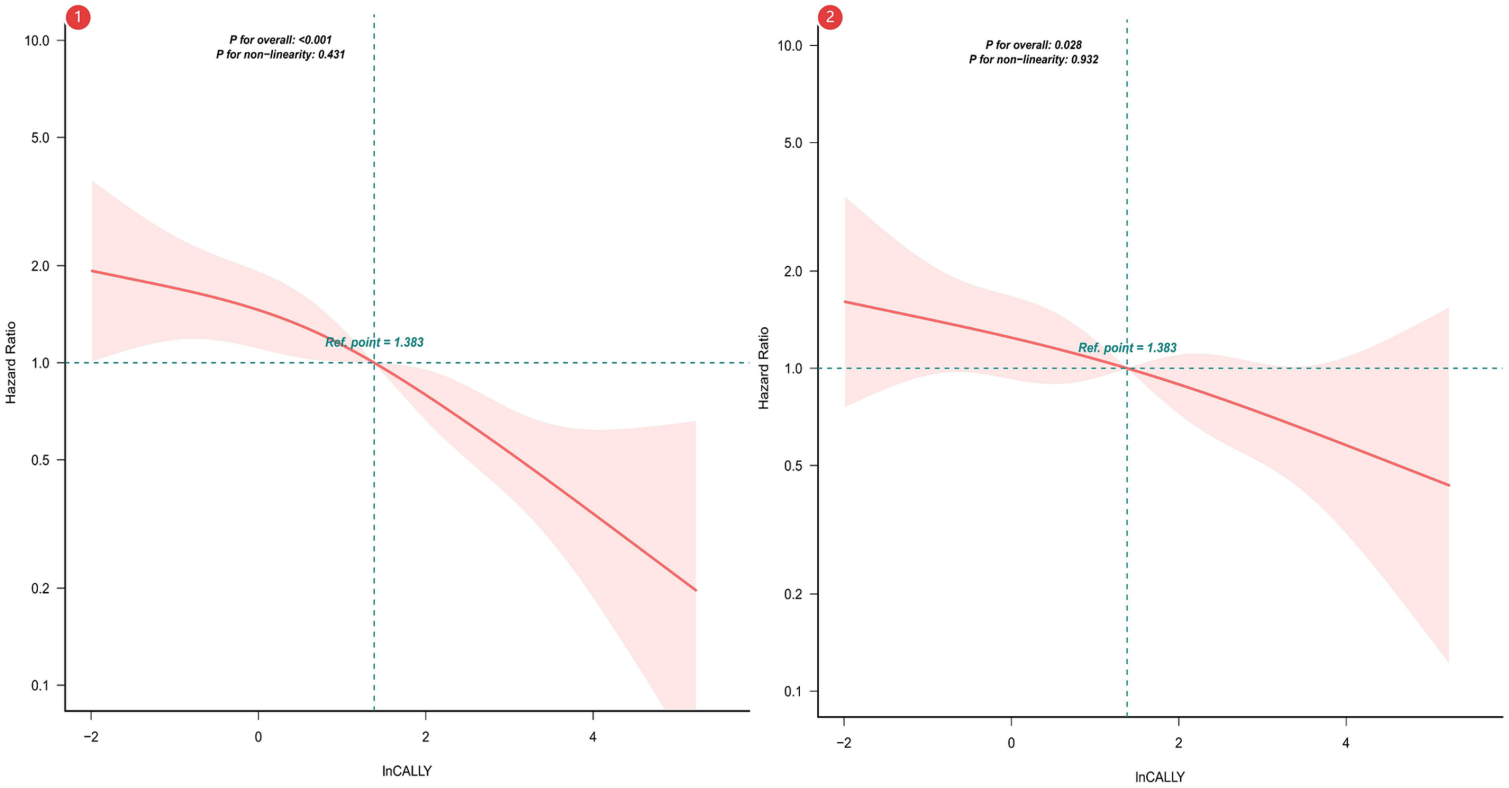
Restricted Cubic Spline Analysis of CALLY Index Association with Overall Survival. ① Unadjusted RCS. ② Adjusted for sex; age; cerebrovascular disease; severe dementia; aspiration pneumonia; ischemic heart disease; hemoglobin levels; percutaneous endoscopic gastrostomy (PEG); total parenteral nutrition (TPN); oral intake recovery status; and daily caloric intake.

To examine the prognostic relevance of the CALLY index, multivariable-adjusted Cox regression models were constructed. Additionally, restricted cubic splines (RCS) modeled the association with overall survival following covariate adjustment. Analyses confirmed an inverse correlation between CALLY index levels and mortality; specifically, increased index values were linked to diminished death risk (Figure 2).

Owing to left-skewed CALLY index distributions, logarithmic conversion was implemented. Quartile stratification of the transformed index (ln-CALLY) facilitated mortality risk assessment in dysphagia cohorts. Table 2 reports adjusted hazard ratios (HR) and 95% confidence intervals for all-cause mortality analyzed via continuous ln-CALLY and quartile-based grouping. Sequential modeling employed four adjustment tiers, spanning unadjusted to maximally adjusted parameters. Within the fully specified Model 4, every log-unit CALLY index elevation manifested a 15% decrease in fatality risk (HR = 0.85, *p* = 0.003). When referenced against Q1 controls, Q4 subjects displayed 78% lower unadjusted mortality (HR = 0.22, *p* < 0.001), retaining statistical significance post-adjustment (HR = 0.44, *p* = 0.005).

**Table 2:** Association between natural log-transformed CALLY index and survival outcomes across multivariable models.

Variable	Total	Event (%)	Crude model		Model 1		Model 2		Model 3		Model 4	
			HR (95%CI)	*P*	HR (95%CI)	*P*	HR (95%CI)	*P*	HR (95%CI)	*P*	HR (95%CI)	*P*
lnCALLY	248	134 (54)	0.74 (0.68–0.82)	<0.001	0.77 (0.7–0.85)	<0.001	0.84 (0.76–0.93)	0.001	0.83 (0.75–0.93)	0.001	0.85 (0.76–0.95)	0.003
Quartiles												
Q1 (≤ − 0.168)	62	48 (77.4)	1 (Ref)		1 (Ref)		1 (Ref)		1 (Ref)		1 (Ref)	
Q2 (−0.168–1.38)	62	38 (61.3)	0.58 (0.38–0.89)	0.012	0.59 (0.39–0.91)	0.017	0.88 (0.55–1.38)	0.568	0.71 (0.45–1.12)	0.144	0.73 (0.46–1.16)	0.179
Q3 (1.38–2.84)	62	29 (46.8)	0.39 (0.24–0.61)	<0.001	0.43 (0.27–0.68)	<0.001	0.55 (0.35–0.89)	0.015	0.49 (0.3–0.79)	0.003	0.56 (0.34–0.9)	0.018
Q4 (>2.84)	62	19 (30.6)	0.22 (0.13–0.37)	<0.001	0.26 (0.15–0.45)	<0.001	0.4 (0.23–0.7)	0.001	0.42 (0.24–0.73)	0.003	0.44 (0.25–0.78)	0.005
*P* for trend				<0.001		<0.001		<0.001		<0.001		0.002

Crude: Unadjusted; Model 1: sex, age; Model 2: sex, age, cerebrovascular disease, severe dementia, aspiration pneumonia, ischemic heart disease, hemoglobin; Model 3: sex, age, cerebrovascular disease, severe dementia, aspiration pneumonia, ischemic heart disease, hemoglobin, PEG, TPN, oral intake recovery; Model 4: sex, age, cerebrovascular disease, severe dementia, aspiration pneumonia, ischemic heart disease, hemoglobin, PEG, TPN, oral intake recovery, daily caloric intake. HR, hazard ratio; CI, confidence interval; Ref, reference.

**Figure 3:**
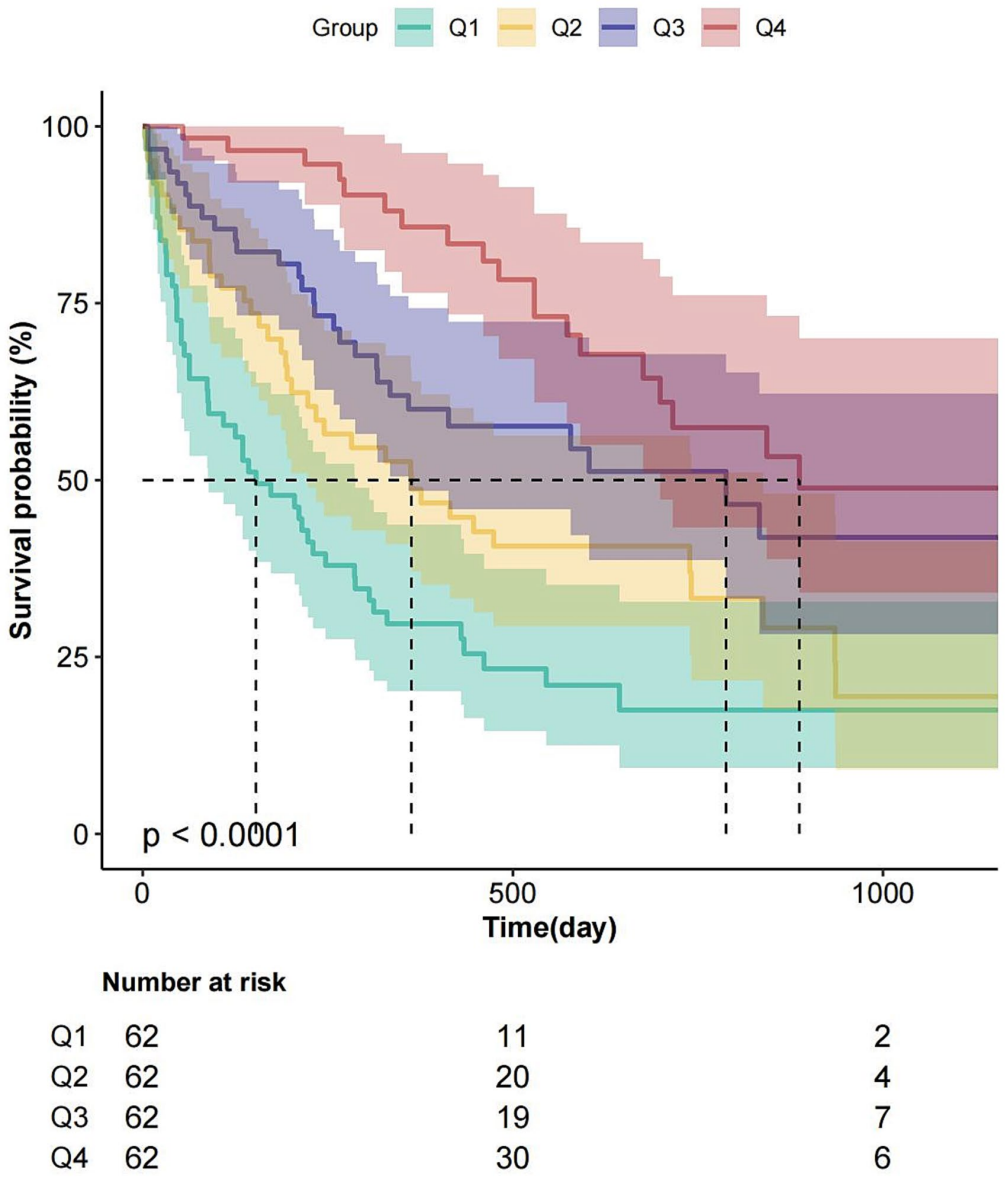
Kaplan–Meier survival analysis stratified by ln-CALLY quartiles. Survival probability (%) according to quartiles of natural log-transformed CALLY index: Q1 (teal; ≤1.38), Q2 (orange; 1.38–2.84), Q3 (dark blue; 2.84–6.79), Q4 (pink; >6.79). Log-rank test revealed significantly different survival curves (*p* < 0.0001), with Q1 (lowest ln-CALLY) exhibiting the poorest survival. Lower table indicates number at risk at 0, 500, and 1,000-day intervals.

The Kaplan–Meier curve shows the relationship between the overall survival of patients with dysphagia in these four groups (Figure 3). Kaplan–Meier analysis revealed progressively longer median survival durations across ascending CALLY quartiles: 153 days (Q1), 362 days (Q2), 785 days (Q3), and 887 days (Q4) (*p* < 0.0001 for trend).

### 3.3 Sensitive analysis

#### 3.3.1 Multiple imputation

To address possible selection bias caused by missing data, multiple imputation was performed by chained equations (MICE). The imputation model included the outcome, all exposure and covariates used in the analytic models, and auxiliary variables predictive of missingness where available. We generated m = 5 imputations and combined estimates using Rubin’s rules (26). We performed a complete-case (available-case) sensitivity analysis; the point estimates and qualitative conclusions were materially similar to the imputed analyses (results presented in Supplementary Table S3).

#### 3.3.2 Excluding early in-hospital deaths and independently adjusting for frailty

To mitigate bias from patients who were imminently dying—who may have both higher inflammatory markers and a different prognosis—we repeated the primary analysis after excluding patients who died within 30 days of admission. The association between the CALLY index and the outcome remained materially unchanged (Supplementary Table S4). Because hospitalized older patients with dysphagia tended to have higher CFS scores and elevated inflammatory markers, we assessed collinearity between CFS and the CALLY index using variance inflation factors and condition indices; no evidence of problematic collinearity was found. Adding CFS to the multivariable Cox model produced a negligible change in the CALLY point estimate (absolute change <0.02) and did not alter the direction or statistical significance of the association (Supplementary Table S6).

#### 3.3.3 Subgroups analysis

**Figure 4:**
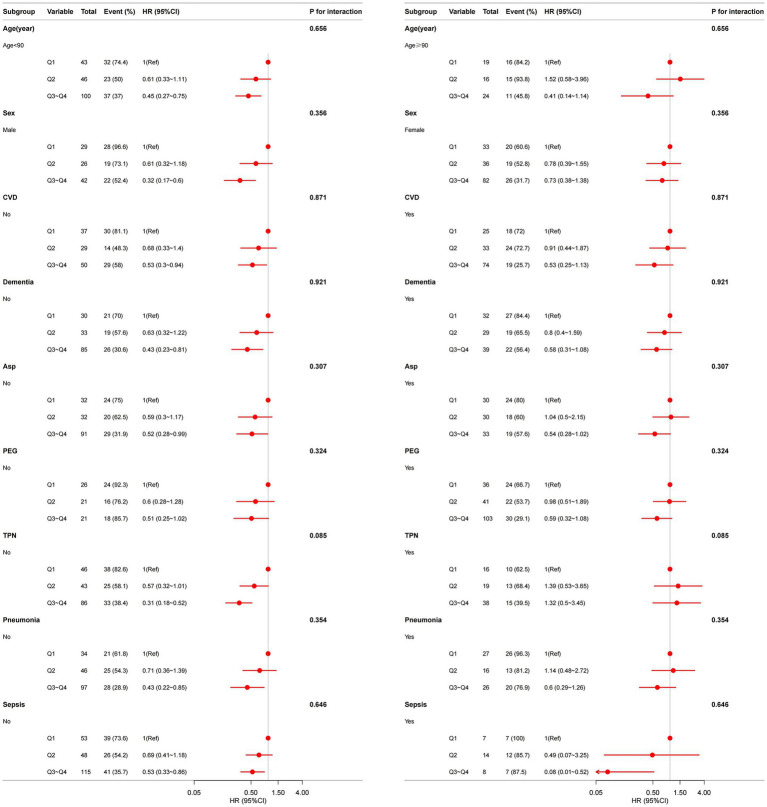
Association between CALLY index and overall survival in older patients with dysphagia in different subgroup. Models are adjusted for sex, age, CVD, severe dementia, Asp., IHD, hemoglobin, PEG, TPN, Oral, daily caloric intake but not adjusted for the stratification variable.

To exclude the possibility that the observed associations were driven by acute inflammatory diseases (pneumonia or sepsis) rather than by the CALLY index, we performed sensitivity analyses stratified by pneumonia and sepsis, tested for interaction, and assessed the consistency and robustness of results across subgroups. There was no statistically significant interaction between CALLY and pneumonia or sepsis (*p* for interaction > 0.05), and the association of CALLY with outcome remained consistent (Figure 4).

#### 3.3.4 Assessing whether the CALLY-outcome association is driven by Alb, CRP, or CAR

To determine whether the observed association merely reflected albumin or CRP alone, we performed comparative analyses. We fitted multivariable Cox proportional hazards models including albumin alone and CRP alone (each model adjusted for the same covariates as the primary analyses) and compared effect estimates with models containing the CALLY index. In our dataset, neither CRP nor albumin was significantly associated with the outcome when included alone in adjusted Cox models, whether modeled continuously or by quartiles, with the exception of CAR in Q4 (Supplementary Table S5). Finally, ROC analyses comparing CALLY, CAR, albumin alone and CRP alone for mortality prediction showed that the CALLY index had the highest AUC (Supplementary Figure S3).

## 4 Discussion

Swallowing represents a complex physiological mechanism necessitating coordinated integration among multiple anatomical systems (27, 28). Pathological compromise at any site along the deglutitive pathway heightens vulnerability to dysphagia. Key susceptible populations include stroke survivors, individuals with neurodegenerative conditions, and head/neck oncology patients (27, 29). Furthermore, even in clinically unaffected individuals, intrinsic aging correlates with elevated dysphagia incidence (27), with advanced age (notably octogenarians) constituting an established risk factor strongly associated with multiple comorbidities (1). Dysphagia in older adults precipitates severe sequelae, substantially impairing general health, nutritional adequacy, and quality of life. Consequently, identifying robust prognostic biomarkers is imperative to facilitate early detection of at-risk individuals and guide personalized management strategies. These markers serve dual functions: estimating survival likelihood while informing targeted interventions, bearing significant implications for enhancing longevity and well-being. This study examined associations between the CALLY index and clinical outcomes in elderly dysphagia patients.

Analyses demonstrated substantially reduced mortality risk among subjects exhibiting higher CALLY index values compared to those with lower levels. The robustness of our findings is supported by multiple sensitivity analyses. Excluding patients who died within 30 days of admission did not materially change the association between the CALLY index and mortality (Supplementary Table S4), indicating that the observed relationship is not driven solely by patients who were imminently dying. After adjustment for CFS, the association between the CALLY index and the outcome remained robust, indicating that the relationship is independent of frailty. Subgroup sensitivity analyses stratified by pneumonia and sepsis showed no significant interaction and yielded consistent associations across groups (Figure 4), further reducing the likelihood that acute inflammatory illness alone accounts for the observed effect. Comparative analyses also indicated that the CALLY index outperformed single biomarkers and CAR (Supplementary Table S5, Supplementary Figure S3).

These results are biologically and methodologically plausible: albumin often shows limited between-patient variability and floor effects in frail, hospitalized cohorts, a single CRP measurement captures only an acute inflammatory snapshot and may not reflect chronic inflammatory–nutritional status, and measurement error/biological variability can attenuate single-marker associations. By integrating complementary domains (inflammation, nutrition and immune status), the CALLY index appears to capture prognostic information not evident from any single component.

Through restricted cubic spline (RCS) analysis, Kaplan–Meier methodology, and Cox proportional hazards modeling, this investigation confirms the CALLY index as an independent predictor of survival in geriatric dysphagia, consistent with prior observations by Jia et al. (14). Excess mortality in elderly dysphagia cohorts demonstrates strong associations with nutritional compromise (30). These results support the hypothesis that systemic inflammation contributes to dysphagia pathophysiology; similar associations between elevated neutrophil-to-lymphocyte and platelet-to-lymphocyte ratios and worse swallowing function have been reported in post intensity modulated radiotherapy nasopharyngeal cancer patients, lending biological plausibility to an inflammation mediated mechanism (31). Malnutrition impairs host defense through diminished leucocyte efficacy, reduced anti-pathogen activity, and dysregulated inflammatory control. It additionally exacerbates oxidative injury and pro-inflammatory mediator production, initiating pathological cascades that promote tissue damage, enteric dysbiosis, and multisystemic decline. Consequently, nutritional optimisation is critical for augmenting immune function, preserving tissue architecture, attenuating inflammation, and reducing mortality. Despite inherent infection risks associated with artificial nutrition support (23), subgroup analyses affirmed the CALLY index’s consistent independent mortality prediction regardless of PEG or TPN administration. Result stability persisted across subgroups stratified by age, sex, severe dementia, and Asp., without significant interaction effects. Future studies should elucidate the influence of temporal CALLY index fluctuations on mortality patterns. A pivotal advantage resides in the index’s foundation on routinely assayed, rapid-turnaround hematological parameters (CRP, Alb, lymphocyte count), incurring minimal additional healthcare expenditure.

Nevertheless, several limitations merit acknowledgement. First, this was a single-center study in an exclusively Japanese population, which may limit generalizability. Second, the analytic sample included patients receiving PEG or TPN and therefore represents a cohort with relatively severe dysphagia; the findings may not apply to patients with milder, conservatively managed disease. Third, as a secondary data analysis, we could not standardize variable selection, sampling times or assay methods. Reliance on a single blood measurement within the first 7 days of admission may introduce selection bias and prevents assessment of temporal changes in Alb, lymphocyte count and CRP. Although we adjusted for multiple important confounders, residual confounding cannot be excluded. Prospective, multicentre studies encompassing a broader spectrum of dysphagia severity and more diverse populations are needed to validate and extend these results.

## 5 Conclusion

The CALLY index independently predicts outcomes in older dysphagia patients, with subgroup assessments detecting no material interaction effects and verifying result consistency.

## Data Availability

The original contributions presented in the study are included in the article/Supplementary material, further inquiries can be directed to the corresponding author/s.

## Footnotes

^1^ http://www.R-project.org
