# Supplementary material for: C-reactive protein-albumin-lymphocyte (CALLY) index predicts overall survival in elderly Japanese patients with dysphagia: a retrospective cohort study
Source: Front Nutr. 2025 Oct 13;12:1681956. doi: 10.3389/fnut.2025.1681956 (PMC12554609; doi:10.3389/fnut.2025.1681956)
Supplement: Supplementary file 1 [file Table_1.DOCX]

**Table S1. The baseline of comparing excluded versus included patients**.

| Variables | Total (n = 253) | Excluded(n = 5) | Included (n = 248) | P-Value |
| --- | --- | --- | --- | --- |
| Age, Mean ± SD | 83.1 ± 9.3 | 83.8 ± 12.6 | 83.0 ± 9.3 | 0.858 |
| Sex, n (%) |  |  |  | 1 |
| Male | 99 (39.1) | 2 (40) | 97 (39.1) |  |
| Female | 154 (60.9) | 3 (60) | 151 (60.9) |  |
| CVD, n (%) |  |  |  | 0.193 |
| No | 120 (47.4) | 4 (80) | 116 (46.8) |  |
| Yes | 133 (52.6) | 1 (20) | 132 (53.2) |  |
| Dementia, n (%) |  |  |  | 1 |
| No | 151 (59.7) | 3 (60) | 148 (59.7) |  |
| Yes | 102 (40.3) | 2 (40) | 100 (40.3) |  |
| Asp, n (%) |  |  |  | 0.654 |
| No | 159 (62.8) | 4 (80) | 155 (62.5) |  |
| Yes | 94 (37.2) | 1 (20) | 93 (37.5) |  |
| IHD, n (%) |  |  |  | **0.046** |
| No | 206 (81.4) | 2 (40) | 204 (82.3) |  |
| Yes | 47 (18.6) | 3 (60) | 44 (17.7) |  |
| Hemoglobin, Mean ± SD | 11.0 ± 2.0 | 10.7 ± 1.5 | 11.0 ± 2.0 | 0.738 |
| Daily calorie intake(kcal), Mean ± SD | 912.1 ± 192.8 | 640.0 ± 290.8 | 917.6 ± 187.1 | **0.001** |
| Oral, n (%) |  |  |  | 0.265 |
| No | 238 (94.1) | 4 (80) | 234 (94.4) |  |
| Yes | 15 ( 5.9) | 1 (20) | 14 (5.6) |  |
| PEG, n (%) |  |  |  | **0.002** |
| No | 73 (28.9) | 5 (100) | 68 (27.4) |  |
| Yes | 180 (71.1) | 0 (0) | 180 (72.6) |  |
| TPN, n (%) |  |  |  | 0.325 |
| No | 180 (71.1) | 5 (100) | 175 (70.6) |  |
| Yes | 73 (28.9) | 0 (0) | 73 (29.4) |  |
| Status, n (%) |  |  |  | 0.38 |
| Alive | 115 (45.5) | 1 (20) | 114 (46) |  |
| Death | 138 (54.5) | 4 (80) | 134 (54) |  |

Abbreviations: PEG percutaneous endoscopic gastrostomy；TPN total parenteral nutrition；CVD cerebrovascular diseases；Dementia severe dementia；Asp aspiration pneumonia；IHD ischemic heart diseases; Oral oral intake recovery.

**Table S2. Assessment of multicollinearity across covariates.**

| Term1 | VIF |
| --- | --- |
| Crude | 1.126 |
| Age | 1.237 |
| Sex | 1.353 |
| CVD | 1.691 |
| Dementia | 1.656 |
| Asp | 1.245 |
| IHD | 1.058 |
| Hb | 1.148 |
| Oral | 1 |
| PEG | 1.283 |
| TPN | 1.081 |
| Daily calorie intake | 1.401 |
| CFS | 1.139 |

Abbreviations: PEG percutaneous endoscopic gastrostomy；TPN total parenteral nutrition；CVD cerebrovascular diseases；Dementia severe dementia；Asp aspiration pneumonia；IHD ischemic heart diseases; Oral oral intake recovery.

**Table S3. Association Between CALLY Index and Survival Outcomes Across Multivariable Models after MICE.**

| **Variable** | **Total** | **Event(%)** | **Crude Model** | | **Model 4** | |
| --- | --- | --- | --- | --- | --- | --- |
|  |  |  | **HR(95%CI)** | ***P*** | **HR(95%CI)** | ***P*** |
| lnCALLY | 253 | 138 (54.5) | 0.75 (0.68~0.82) | <0.001 | 0.84 (0.75~0.94) | 0.002 |
| **Quartiles** |  |  |  |  |  |  |
| Q1 | 63 | 49 (77.8) | 1(Ref) |  | 1(Ref) |  |
| Q2 | 63 | 38 (60.3) | 0.56 (0.37~0.86) | 0.008 | 0.73 (0.46~1.16) | 0.184 |
| Q3 | 63 | 32 (50.8) | 0.43 (0.27~0.67) | <0.001 | 0.52 (0.33~0.84) | 0.008 |
| Q4 | 64 | 19 (29.7) | 0.2 (0.12~0.35) | <0.001 | 0.44 (0.25~0.78) | 0.005 |
| P for trend |  |  |  | <0.001 |  | 0.001 |

Notes: Crude: Unadjusted;Model 4: sex, age, cerebrovascular disease, severe dementia, aspiration pneumonia, ischemic heart disease, hemoglobin, PEG, TPN, oral intake recovery, daily caloric intake. Abbreviations: HR hazard ratio; CI confidence interval; Ref reference

**Table S4. Associations between the log-transformed CALLY index and overall survival in patients with survival of more than 30 days.**

| **Variable** | **Total** | **Event(%)** | **Crude Model** | | **Model 1** | | **Model 2** | | **Model 3** | | **Model 4** | |
| --- | --- | --- | --- | --- | --- | --- | --- | --- | --- | --- | --- | --- |
|  |  |  | **HR(95%CI)** | ***P*** | **HR(95%CI)** | ***P*** | **HR(95%CI)** | ***P*** | **HR(95%CI)** | ***P*** | **HR(95%CI)** | ***P*** |
| lnCALLY | 228 | 116(50.9) | 0.77(0.7~0.85) | <0.001 | 0.8(0.72~0.89) | <0.001 | 0.86(0.77~0.95) | 0.005 | 0.85(0.76~0.95) | 0.005 | 0.86(0.76~0.96) | 0.01 |
| **Quartiles** |  |  |  |  |  |  |  |  |  |  |  |  |
| Q1 | 48 | 36 (75) | 1(Ref) |  | 1(Ref) |  | 1(Ref) |  | 1(Ref) |  | 1(Ref) |  |
| Q2 | 57 | 31(54.4) | 0.48(0.3~0.78) | 0.003 | 0.55(0.34~0.89) | 0.015 | 0.76(0.45~1.28) | 0.297 | 0.61(0.36~1.03) | 0.064 | 0.6(0.35~1.01) | 0.055 |
| Q3 | 61 | 30 (49.2) | 0.43(0.27~0.7) | 0.001 | 0.51(0.32~0.84) | 0.008 | 0.63(0.38~1.04) | 0.069 | 0.54(0.33~0.89) | 0.016 | 0.58(0.35~0.97) | 0.038 |
| Q4 | 62 | 19 (30.6) | 0.22(0.12~0.38) | <0.001 | 0.28(0.16~0.49) | <0.001 | 0.41 (0.22~0.74) | 0.003 | 0.41(0.22~0.75) | 0.004 | 0.43(0.23~0.78) | 0.006 |
| P for trend |  |  |  | <0.001 |  | <0.001 |  | 0.002 |  | 0.002 |  | 0.006 |

Notes: Crude: Unadjusted;Model 1: sex, age;Model 2: sex, age, cerebrovascular disease, severe dementia, aspiration pneumonia, ischemic heart disease, hemoglobin;Model 3: sex, age, cerebrovascular disease, severe dementia, aspiration pneumonia, ischemic heart disease, hemoglobin, PEG, TPN, oral intake recovery;Model 4: sex, age, cerebrovascular disease, severe dementia, aspiration pneumonia, ischemic heart disease, hemoglobin, PEG, TPN, oral intake recovery, daily caloric intake; Abbreviations: HR hazard ratio; CI confidence interval; Ref reference

**Table S5. Associations between three nutrition index and overall survival in patients.**

| **Variable** | **Total** | **Event_%** | **Crude Model** | | **Model 4** | |
| --- | --- | --- | --- | --- | --- | --- |
|  |  |  | **HR (95%CI)** | ***P*** | **HR (95%CI)** | ***P*** |
| **lnCALLY** | 253 | 138 (54.5) | 0.75 (0.68~0.82) | <0.001 | 0.84 (0.75~0.94) | 0.002 |
| Q1 | 63 | 49 (77.8) | 1(Ref) |  | 1(Ref) |  |
| Q2 | 63 | 38 (60.3) | 0.56 (0.37~0.86) | 0.008 | 0.73 (0.46~1.16) | 0.184 |
| Q3 | 63 | 32 (50.8) | 0.43 (0.27~0.67) | <0.001 | 0.52 (0.33~0.84) | 0.008 |
| Q4 | 64 | 19 (29.7) | 0.2 (0.12~0.35) | <0.001 | 0.44 (0.25~0.78) | 0.005 |
| P for trend | 253 | 138 (54.5) | 0.61 (0.52~0.71) | <0.001 | 0.75 (0.63~0.89) | **0.001** |
| **Alb** | 253 | 138 (54.5) | 0.38 (0.29~0.51) | <0.001 | 0.8 (0.55~1.18) | 0.263 |
| Q1 | 60 | 43 (71.7) | 1(Ref) |  | 1(Ref) |  |
| Q2 | 54 | 36 (66.7) | 0.81 (0.52~1.26) | 0.342 | 0.76 (0.47~1.23) | 0.265 |
| Q3 | 75 | 42 (56) | 0.55 (0.36~0.84) | 0.005 | 0.88 (0.55~1.41) | 0.592 |
| Q4 | 64 | 17 (26.6) | 0.2 (0.11~0.36) | <0.001 | 0.72 (0.36~1.44) | 0.354 |
| P for trend | 253 | 138 (54.5) | 0.63 (0.54~0.73) | <0.001 | 0.92 (0.75~1.13) | **0.446** |
| **CRP** | 253 | 138 (54.5) | 1.1 (1.04~1.15) | <0.001 | 1.05 (0.99~1.11) | 0.109 |
| Q1 | 63 | 22 (34.9) | 1(Ref) |  | 1(Ref) |  |
| Q2 | 63 | 29 (46) | 1.47 (0.85~2.57) | 0.171 | 1.65 (0.92~2.96) | 0.09 |
| Q3 | 63 | 41 (65.1) | 2.56 (1.52~4.3) | <0.001 | 1.53 (0.85~2.75) | 0.155 |
| Q4 | 64 | 46 (71.9) | 3.39 (2.04~5.66) | <0.001 | 1.96 (1.13~3.41) | **0.017** |
| P for trend | 253 | 138 (54.5) | 1.52 (1.3~1.77) | <0.001 | 1.21 (1.02~1.43) | 0.029 |

Notes: Crude: Unadjusted; Model 4: sex, age, cerebrovascular disease, severe dementia, aspiration pneumonia, ischemic heart disease, hemoglobin, PEG, TPN, oral intake recovery, daily caloric intake; Abbreviations: HR hazard ratio; CI confidence interval; Ref reference

**Table S6. Additional adjusted for CFS in Multivariable Models.**

| **Variable** | **Total** | **Event(%)** | **Crude Model** | | **Model 4** | |
| --- | --- | --- | --- | --- | --- | --- |
|  |  |  | **HR(95%CI)** | ***P*** | **HR(95%CI)** | ***P*** |
| lnCALLY | 248 | 134 (54) | 0.74 (0.68~0.82) | <0.001 | 0.84 (0.76~0.94) | 0.001 |
| **Quartiles** |  |  |  |  |  |  |
| Q1 | 62 | 48 (77.4) | 1(Ref) |  | 1(Ref) |  |
| Q2 | 62 | 38 (61.3) | 0.58 (0.38~0.89) | 0.012 | 0.8 (0.51~1.27) | 0.342 |
| Q3 | 62 | 29 (46.8) | 0.39 (0.24~0.61) | <0.001 | 0.55 (0.34~0.89) | 0.015 |
| Q4 | 62 | 19 (30.6) | 0.22 (0.13~0.37) | <0.001 | 0.43 (0.24~0.76) | 0.003 |
| P for trend |  |  |  | <0.001 |  | 0.001 |

Notes: Crude: Unadjusted;Model 4: sex, age, cerebrovascular disease, severe dementia, aspiration pneumonia, ischemic heart disease, hemoglobin, PEG, TPN, oral intake recovery, daily caloric intake and CFS. Abbreviations: HR hazard ratio; CI confidence interval; Ref reference

**
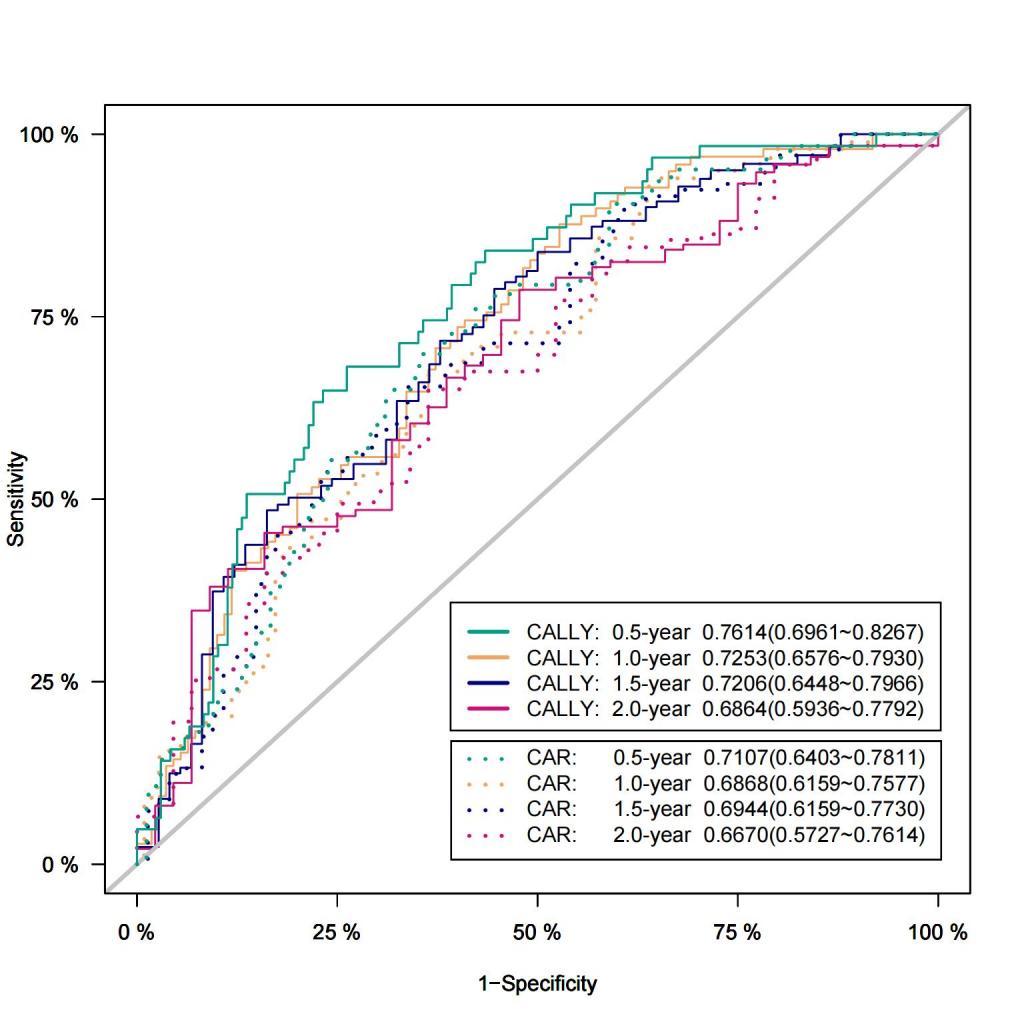
**

**Figure S1.Time dependent receiver operating characteristic curves comparing CALLY and CAR for predicting mortality.**

**
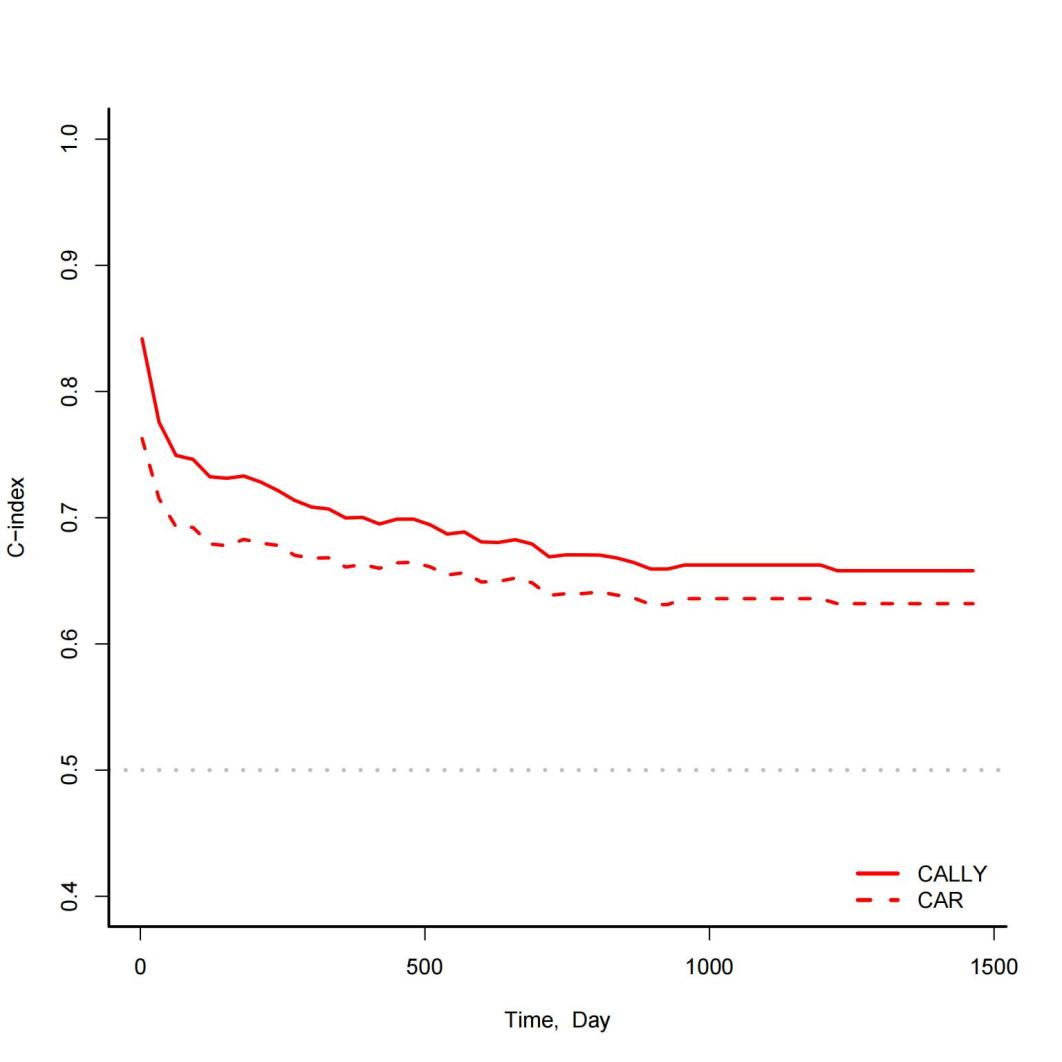
**

**Figure S2. Time‑dependent concordance index (C‑index) for CALLY and CAR over the follow‑up period.**


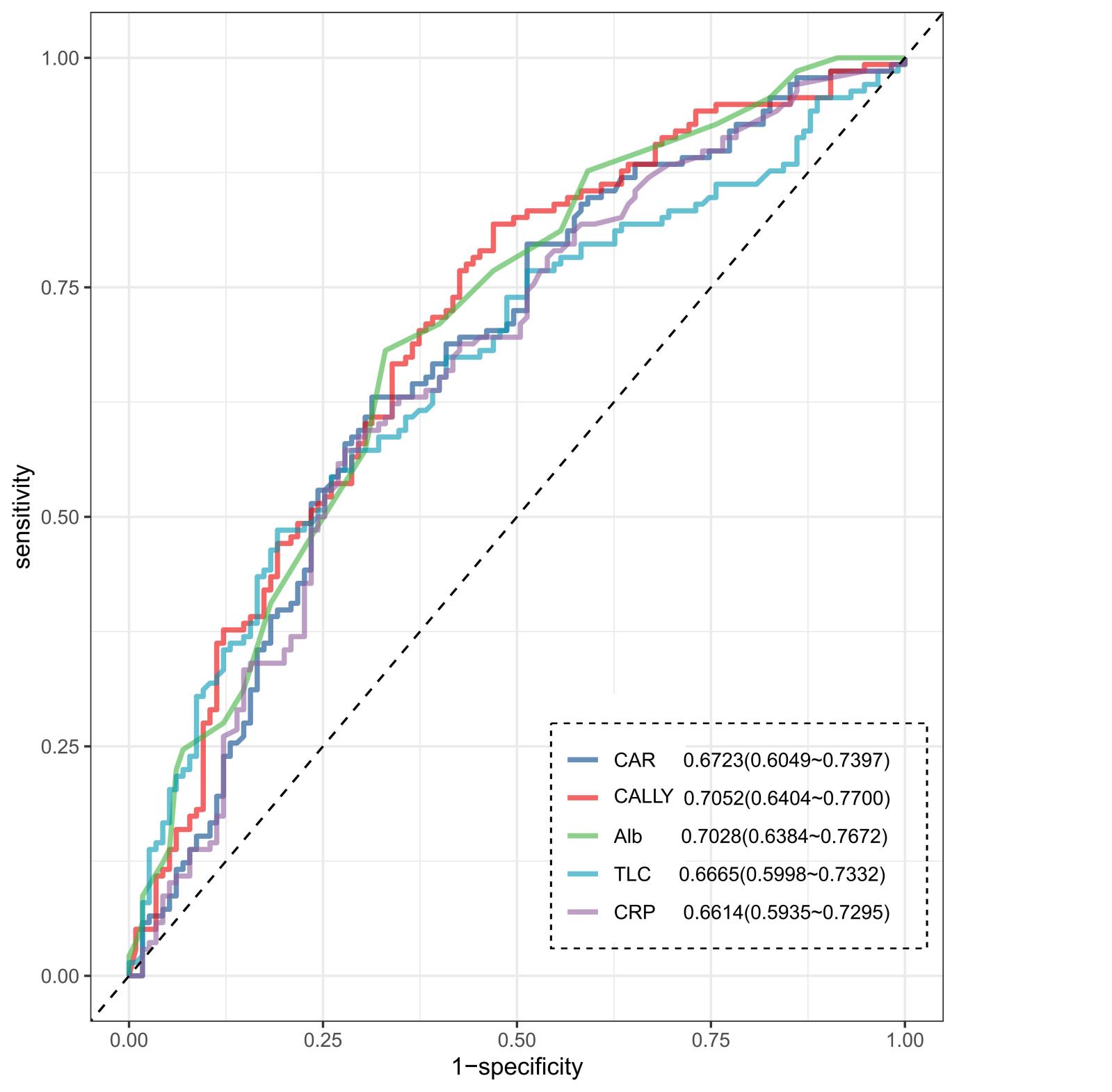


**Figure S3.ROC curves comparing five markers for predicting mortality**
